# Measurement of Water Leaving Reflectance Using a Digital Camera Based on Multiple Reflectance Reference Cards

**DOI:** 10.3390/s20226580

**Published:** 2020-11-18

**Authors:** Min Gao, Junsheng Li, Fangfang Zhang, Shenglei Wang, Ya Xie, Ziyao Yin, Bing Zhang

**Affiliations:** 1 Key Laboratory of Digital Earth Science, Aerospace Information Research Institute, Chinese Academy of Sciences, Beijing 100094, China; gaomin@radi.ac.cn (M.G.); zhangff07@radi.ac.cn (F.Z.); wangsl@radi.ac.cn (S.W.); 3001200119@cugb.edu.cn (Y.X.); yinzy@aircas.ac.cn (Z.Y.); zb@radi.ac.cn (B.Z.); 2 College of Resources and Environment, University of Chinese Academy of Sciences, Beijing 100049, China; 3 School of Electronic, Electrical and Communication Engineering, University of Chinese Academy of Sciences, Beijing 100049, China; 4 School of Earth Sciences and Resources, China University of Geosciences (Beijing), Beijing 100083, China

**Keywords:** digital camera, citizen science, water leaving reflectance, remote sensing reflectance

## Abstract

With the development of citizen science, digital cameras and smartphones are increasingly utilized in water quality monitoring. The smartphone application HydroColor quantitatively retrieves water quality parameters from digital images. HydroColor assumes a linear relationship between the digital pixel number (DN) and incident radiance and applies a grey reference card to derive water leaving reflectance. However, image DNs change with incident light brightness non-linearly, according to a power function. We developed an improved method for observing and calculating water leaving reflectance from digital images based on multiple reflectance reference cards. The method was applied to acquire water, sky, and reflectance reference card images using a Cannon 50D digital camera at 31 sampling stations; the results were validated using synchronously measured water leaving reflectance using a field spectrometer. The R^2^ for the red, green, and blue color bands were 0.94, 0.95, 0.94, and the mean relative errors were 27.6%, 29.8%, 31.8%, respectively. The validation results confirm that this method can derive accurate water leaving reflectance, especially when compared with the results derived by HydroColor, which systematically overestimates water leaving reflectance. Our results provide a more accurate theoretical foundation for quantitative water quality monitoring using digital and smartphone cameras.

## 1. Introduction

With the rapid development of modern big data and communication technologies, environmental quality monitoring has entered the era of crowdsourcing big data [1,2,3]. As citizen participation increases, citizen science becomes an important source in collecting crowdsourcing big data to provide more valuable scientific data [4]. Citizen science refers to the involvement of the community and collecting data by these non-professionals in organized research endeavors [5]. The participation of citizen scientists in environmental data collection can complement traditional monitoring methods and Earth observation methods, because it has many potential advantages, such as reduced monitoring costs, increased data coverage, enhanced support for decision-making, and enhanced potential for knowledge co-creation [6,7,8]. Due to the outstanding value of citizen science in data collection, including temporal resolution and spatial scope, it has been recognized as a support for the United Nations Sustainable Development Goals (SDG) [9,10]. Especially in developing countries where the availability of data and the financial resources are limited, such an approach helps to expand the monitoring network in a cost-effective way [11].

Citizen Science is contributing to Sustainable Development Goal indicator 6.3.2 “Clean Water and Sanitation” [12]. As a low-cost and powerful citizen science tool, smartphones play an important role in water quality data collection [11,13]. The images taken by a smartphone exhibit a high spatial resolution, which is conducive to the monitoring of small water bodies. Moreover, they are not affected by cloud coverage [14,15]. More and more projects using smartphone application (App) as a method of collecting water quality data are being established. CyanoAlert App was cooperatively developed by U.S. Environmental Protection Agency (EPA) and EU H2020 CyanoAlert project. It can provide users with satellite-based water quality information, and users can also report their surrounding water quality information to provide decision-making for others. It is a very useful tool for citizen science educators in water quality. FreshWaterWatch (FWW) was developed by Multiscale Observation Networks for Optical monitoring of Coastal waters, Lakes and Estuaries (MONOCLE) and Earthwatch Europe. This is a water quality monitoring program that aims to understand the drivers and causes of freshwater degradation to better manage and protect the world’s freshwater resources. Participants test for indicators of water quality, and record contextual observations like water color, algal growth, and upload database through FWW App. The UK Centre for Ecology & Hydrology developed the Bloomin’Algae App to report blue-green algae blooms in lakes, reservoirs, and rivers by capturing images and utilizing the phone positioning function, to assist decision-makers in monitoring and controlling water blooms (https://www.ceh.ac.uk/algal-blooms/bloomin-algae). The European Citlops project developed the EyeonWater App, in which the Forel-Ule Index (water color parameter) is obtained by comparing smartphone images [16,17,18].

Previous projects have primarily focused on obtaining water images and related auxiliary information, but have not used water images to retrieve water quality parameters. However, Leeuw et al. [19,20] developed the smartphone App HydroColor, which uses the smartphone camera as a three-band radiometer to quantitatively retrieve water quality parameters from water images. According to observation angle information, users typically take an 18% reflectance gray card, water, and sky images. The clipped images are then used to calculate the water leaving reflectance of the RGB bands and to estimate the water turbidity. Water leaving reflectance is the ratio of water leaving radiance and water surface downward irradiance, which is often called remote sensing reflectance. Water leaving reflectance reflects information on the substances within the water body and is one of the key parameters in retrieving water quality by remote-sensing techniques. HydroColor can be downloaded from App stores and has been applied and validated by several studies. Malthus et al. [21] used HydroColor to collect water images from 32 sampling stations in eastern Australia to calculate water leaving reflectance, which was compared with in situ measurements. The accuracy of HydroColor was lower when the surrounding water environment was complex. Yang et al. [22] applied HydroColor to the coast of western Canada, and data were collected by both trained and untrained citizens. The results demonstrated that HydroColor is more effectively utilized by trained individuals and that its accuracy is higher when there is no cloud cover.

Although HydroColor allows for the quantitative application of water quality parameters retrieved by a digital camera, it does have limitations. This assumes that there is a linear relationship between camera-measured digital pixel number (DN) values and the incident light radiance, which is the basis of the water leaving reflectance derivation and water quality parameter inversion. However, studies have shown that the linear relationship hypothesis between the DN and incident light radiance in HydroColor is not accurate [21,23,24]. Therefore, the purpose of this study was to develop a method to simulate the nonlinear relationship between the DN and incident light radiance by multiple reflectance reference cards, thereby deriving water leaving reflectance from the nonlinear corrected DN in digital images. Our results provide a more accurate theoretical foundation for quantitative water quality monitoring using digital and smartphone cameras.

## 2. Digital Camera Optical Response Analysis

To accurately calculate water leaving reflectance using digital images, the optical response of the digital camera should first be analyzed, including the relationship between the DN and exposure parameters and the relationship between DN and surface reflectance. A Canon 50D digital camera, which can conveniently control the exposure parameters, was selected for analysis.

### 2.1. Response Analysis of Digital Image DN with Exposure Parameters

When a digital camera is used to take images for calculating water leaving reflectance, images of a reference card, water, and sky must all be taken. Water and sky images cannot be taken by the same camera simultaneously; therefore, fixed exposure parameters of the reference card are used to make the images easily comparable. However, this method may lead to two problems: (1) not all fully automatic cameras support the adjustment of exposure parameters, making this method unusable; (2) the reflectance of the water and sky are substantially different, and using a low-exposure parameter to avoid overexposing sky images will make water images dark, and may increase the noise in water images.

**Figure 1:**
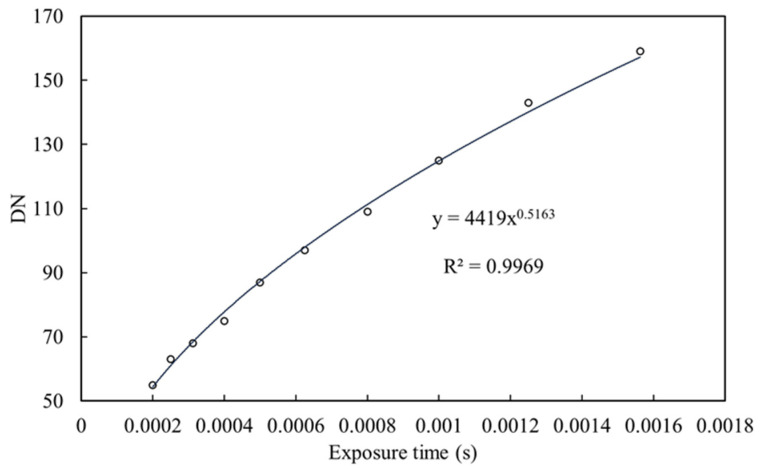
Scatterplot and fitting line of red band digital pixel number (DN) as a function of exposure time of a gray reference card with a fixed aperture (1/7.1) and ISO (125).

To resolve these problems, we analyzed the DN responses of digital images to the changes in exposure parameters to determine if there is a change rule. Then, we could normalize the DN of images obtained under varying exposure parameters. Exposure parameters include: exposure time, aperture, and the focal plane’s sensitivity to light, which is controlled by the International Standards Organization (ISO) setting. We fixed the aperture and ISO, and changed the exposure time to capture the resulting changes in image DN. We captured a grey reference card with a fixed aperture of f/7.1, ISO 125, and changing exposure times (1/640, 1/800, 1/1000, 1/1250, 1/1600, 1/2000, 1/2500, 1/3200, 1/4000, and 1/5000). The changes in the DN of the red band of the obtained reference card image as a function of the exposure time are shown in Figure 1. The optical responses of the green and blue bands of digital images are consistent with that of the red band and are not discussed further.

According to Figure 1, the relationship between the DN of an object and the exposure time is not linear but a power function. If we want to compare the DNs of images taken at various exposure times, we must take at least three images of the same object at different exposure times. Afterward, we can fit the curve function of the exposure time and DN, and then normalize the DNs based on exposure time.

This normalization correction method is unsuitable for fully automatic cameras where exposure parameters cannot be controlled. For cameras that can manually set the exposure parameters, at least three images with different exposure times should be taken of the same object, including at least one image with a short exposure time. The image DNs are affected by noise and the fitting curve function also introduces errors. The superposition of noise and error brings uncertainty into this method.

Considering all factors, the optimal approach is to take images of reference cards, water, and sky using uniform fixed exposure parameters. To minimize the noise impact of water images, we set a longer exposure time, resulting in high but not saturated DNs of the reference card and sky images (such as >200, and <250).

### 2.2. Response Analysis of Digital Image DN with Reflectance

Based on the analysis in Section 2.1, the same exposure parameters were used for each of the three images (reference cards, water, and sky). The DNs of this approach are consistent with those obtained by taking three types of objects in the same image. We must know the relationship between the reflectance of different objects in the same image and their DNs to calculate the reflectance of water and sky based on the reference card reflectance and water leaving reflectance.

When the DN of an image changes linearly with object reflectance and the fitting line passes through the origin (the intercept is 0), the gain of the fitting line can be calculated using one reference card, and the reflectance of the water and sky are the results of DN multiplied by the gain. However, when the DN of the image changes linearly with the reflectance of the object, but the fitting line does not pass through the origin, two reflectance reference cards must be used to calculate the gain and offset of the fitting line, and then the DNs of the water and sky can be calculated as the reflectance. When the DN of the image changes nonlinearly with the object reflectance, at least three reflectance reference cards are required to fit the curve of the DN versus reflectance, and then the DNs of the water and sky can be calculated as the reflectance.

**Figure 2:**
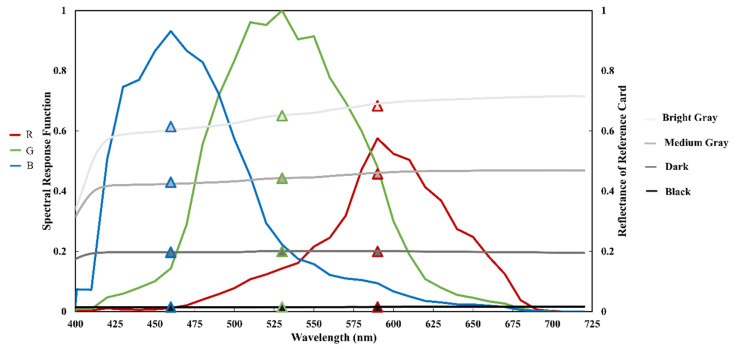
Spectral response functions of the Canon 50D RGB bands (red, green, and blue curves), the reflectance curves of the four reference cards (bright gray, medium gray, dark gray, and black curves), and results of the equivalent reflectance of the reference cards in the Canon 50D RGB bands (triangles).

We purchased four reference cards with different reflectance (bright gray, medium gray, dark gray, and black) from Anhui Institute of Optics and Fine Mechanics, Chinese Academy of Sciences. These reference cards are made by high-strength fabric coated with spectral neutral coating to form a flat diffuse and Lambertian surface, which are originally used for in-flight absolute radiometric calibration for air-borne and space-borne remote sensors [25]. The reflectance of the four reference cards were measured 90° vertically by a spectrometer in laboratory before usage, which are shown as the gray/black lines in Figure 2.

Each camera has three bands (RGB), and each band exhibits a spectral response function. The spectral response functions of each RGB band of the Canon 50D are displayed as red, green, and blue curves in Figure 2 [26]. Using Equation (1), we can calculate the equivalent reflectance of the reference card in each RGB band of the Canon 50D

(1) $$\mathrm{Rrs}\left(\lambda_{i}\right)=\;\frac{\int_{\lambda_{\min}}^{\lambda_{\max}}\mathrm{Rrs}\left(\lambda \right)\times f\left(\lambda \right)d\lambda }{\int_{\lambda_{\min}}^{\lambda_{\max}}f\left(\lambda \right)d\lambda }$$

where Rrs (λ) is the incoming spectrum; f(λ) is the spectral response function of the RGB band of the digital camera; and λ_min_ and λ_max_ are the minimum and maximum spectral response wavelengths in the RGB band, respectively. The calculated reflectance of each reference card in the RGB bands are displayed as triangles in Figure 2.

**Figure 3:**
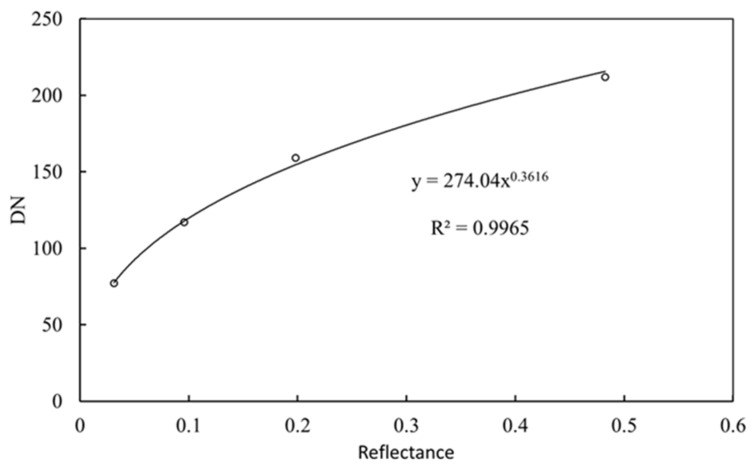
Scatterplot and fitting line of reflectance of the four reference cards and corresponding digital pixel numbers (DNs) in the red band. The relationship between the DNs and their equivalent reflectance is nonlinear, and the R^2^ fitted by power function reaches 0.9965.

The camera was used to take a picture, and optimal exposure parameters were set to avoid overexposing the light gray card (255), while preventing the black card from being too dark in the image. The R band curve of the DN from the reflectance of the four reference cards was obtained (Figure 3). The DN curves in the G and B bands of the digital images with the reflectance of reference cards were consistent with that of the R band.

According to the sRGB color space proposal published in 1996 [27], the response of the human eye to incident light intensity is nonlinear. To simulate the nonlinear response, the DN of a digital image is also set as a nonlinear response to incident light intensity (i.e., gamma correction is performed, which is a power function correction). Presently, mainstream cameras on the market all perform gamma corrections for input values through power operation, which explains why the relationship between the DN and reflectance of digital images displayed in Figure 3 can be fitted by a power function.

In summary, as the relationship between DN and reflectance is nonlinear, it is necessary to use at least three reflectance reference cards to fit the reflectance (Ref) and DN curve. Then, the curve can be used to calculate the reflectance of water and sky and, subsequently, the water leaving reflectance.

## 3. Derivation of Water Leaving Reflectance Based on Digital Images

### 3.1. Photographing Method for Obtaining Water Leaving Reflectance

To calculate the water leaving reflectance of the water body through photography, reasonable observation geometry must be designed to avoid the influence of the sun and other factors. Optimal exposure parameters and reference cards were set according to the results of the digital camera optical response analysis in Section 2.

#### 3.1.1. Observation Geometry Design

The problems encountered in obtaining water leaving radiation using a camera are the same as those of ordinary field spectrometers, such as avoiding the influence of solar glint and shore reflectance. Therefore, the normal spectral observation geometry above the water surface in ocean optic protocols [28] can also be used when a camera is used to obtain water leaving reflectance. The water image is approximately captured at 30°–45° from nadir and 135° from the plane of the sun. The sky image is approximately captured at 135°–150° from nadir and 135° from the plane of the sun. The reference card image is captured at 90° vertically downward, with the reference cards at a horizontal level without shadowing.

#### 3.1.2. Exposure Settings

Based on the results in Section 2.1, the exposure parameters (ISO, aperture, exposure time) should be fixed to take the images of the reference card, water, and sky in sequence. When setting the exposure parameters, we try to get high (near to 255) but not saturated (equal to 255) DN values of the brightest reference card and the sky. When taking images, the light must be stable; when there are clouds around the sun causing variations in light, photographing should be delayed or additional photos should be taken.

#### 3.1.3. Reference Card Usage

According to the results in Section 2.2, at least three reflectance reference cards are required to fit the relationship between the surface reflectance and its DNs to calculate the reflectance of water and sky and, subsequently, the water leaving reflectance. In the following experiments, four reflectance reference cards (Figure 2) were used.

### 3.2. Water Surface Experiments for Obtaining Water Leaving Reflectance

In 2018 and 2019, we conducted three water surface experiments at 31 sampling stations of Taihu Lake and Yuqiao Reservoir, and the metadata of these experiments are shown in Table 1. During the three days of the experiments, the weather was sunny, without cloud near the sun. The experiments were carried out during 9:30–15:45 h local time, and the solar zenith angles range from 16° to 66°.

**Figure 4:**
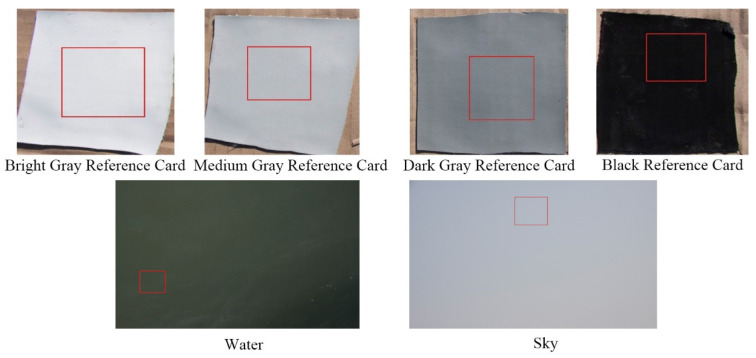
Sample images of four reference cards, water, and sky taken by a digital camera. The red box indicates the position of the uniform area after clipping.

The methods described in Section 3.1 were used to take the images of the four reflectance reference cards, water, and sky. An example of these images is shown in Figure 4.

Immediately after the images were taken, portable field spectrometer (Analytical Spectral Device, Inc., Boulder, CO, USA, FieldSpec^®^) was used at each water surface sampling station based on the proposed ‘above water method’ [28]. Using a spectrometer, the radiance of a 30 × 30 cm^2^ reference panel (L_p_), total radiance of water body (L_sw_), and downward radiance of sky (L_sky_) was measured. The spectra of total radiance of water body were measured 10 times at each sampling station. The outliers in the 10 spectra are mostly those affected by sun-glint, and will be excluded from the datasets. The rest of the spectra will be averaged to calculate the L_sw_. The equation for calculating remote sensing reflectance (Rrs) from the measured spectral data is as follows [29,30]

Rrs = (L_sw_ − r_sky_ × L_sky_)/(L_p_ × π/R_p_) (2)

where r_sky_ is the fraction of the skylight reflected by the water surface, which depends on the position of the sun, observation geometry, and water surface roughness. In the ‘above water method’, the viewing direction is about 40° from nadir and the viewing azimuth is about 135° from the plane of the sun. For this observation geometry, and for relatively low wind speed during the three experiments, r_sky_ ≈ 0.028 was regarded to be acceptable [29]. R_p_ is the reflectance of the reference panel, which was calibrated in the laboratory.

**Figure 5:**
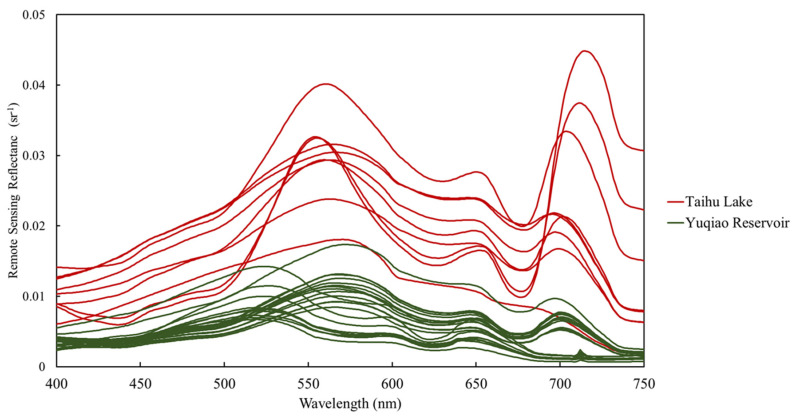
Remote sensing reflectance spectra of water surfaces measured with a field spectrometer at Taihu Lake and the Yuqiao Reservoir.

The Rrs results of the 31 sampling stations, which were calculated from the water, sky, and reference panel measurements using Equation (2), are shown in Figure 5. These Rrs spectra were then used in Equation (1), along with the spectral response functions of each RGB band of the Canon 50D, to determine the equivalent Rrs of each Canon 50D RGB band (Rrs_m_), which can be used as the true values to validate the Rrs calculated from synchronously obtained water surface images.

### 3.3. Water Leaving Reflectance Derivation from Digital Images Based on Multiple Reflectance Reference Cards

#### 3.3.1. Water, Sky, and Reference Card Photography

First, the water images were cut to avoid sun glint, shadows, and floater areas. Then, sky images were cut and the area opposite to the zenith angle of the water observation were selected. Finally, each reference card was cut. After clipping, the median values of the water, sky, and clipped area of each reference card image were calculated.

#### 3.3.2. Calculation of Water and Sky Reflectance

According to the results in Section 2.2, the power function model can be used to fit the curve of the relationship between reflectance and DN using the reflectance of at least three reference cards and the corresponding cut image DN median value

Ref = a × DN^b^ (3)

where the coefficients a and b were obtained by fitting Equation (3) with the reflectance of the reference card and its DN.

Based on the fitting function, the median DN of the clipped water images (DN_w_) and median DN of the cropped sky image (DN_s_) were used to calculate the reflectance of the water body (Ref_w_) and sky (Ref_s_).

#### 3.3.3. Calculation of Water Leaving Reflectance

The water reflectance (Ref_w_) and sky reflectance (Ref_s_) were introduced into the calculation equation of Rrs, and the equation derived from Equation (2) for calculating the water leaving reflectance was obtained as follows

Rrs = (Ref_w_ – (r_sky_ × Ref_s_))/π (4)

### 3.4. Water Leaving Reflectance Derivation from Digital Images Based on the Method in HydroColor

The experiment results in Section 2.2 have revealed the problem of the linear response hypothesis in the method used in HydroColor. We want to analyze the effect of the linear response hypothesis further, quantitatively of derivation of water leaving reflectance. We did not directly utilize HydroColor App in a smartphone to collect images for calculating water leaving reflectance during the three experiments, because it may have inconsistency with the images taken by the Cannon 50D camera, due to the differences in observation geometry, exposure parameters, and signal-to-noise. Instead, we applied the method in HydroColor to the same images taken by the Cannon 50D camera. By this means, the differences in the Rrs results derived by the method in HydroColor and this study can be used to analyze the effect of the linear response hypothesis in HydroColor.

HydroColor assumes that the DN changes linearly with the incident light brightness and uses an 18% reflectance gray card to calculate water leaving reflectance as follows

Rrs = (L_t_ – ρ × L_s_)/(L_c_ × π/Ref_c_) (5)

where L_t_, L_s_, and L_c_ are the DNs of the water surface, sky, and gray card, respectively; Ref_c_ is the reflectance of the gray card; ρ is the fraction of skylight reflected by the water surface.

In this study, there was one gray card with ~18% reflectance among the four reflectance reference cards. Using this gray card, the Rrs of the 31 sampling stations were calculated using Equation (5).

### 3.5. Accuracy Evaluation of Water Leaving Reflectance Derived from Digital Images

To evaluate the accuracy of the calculation of Rrs, the root mean square error (RMSE), mean relative error (MRE), square of the correlation (R^2^), and the mean ratio [31] were used as evaluation indices

(6) $$\mathrm{RMSE}=\;\sqrt{\sum_{i=1}^{N}{\left({\mathrm{Rrs}}_{p}-{\;\mathrm{Rrs}}_{m}\right)}^{2}/N}$$

(7) $$\mathrm{MRE}=\;\sum_{i=1}^{N}\left(\left|{\mathrm{Rrs}}_{p}-{\;\mathrm{Rrs}}_{m}\right|/{\mathrm{Rrs}}_{m}\right)/N$$

(8) $$\mathrm{Ratio}=\left(\sum_{i=1}^{N}\frac{{\mathrm{Rrs}}_{p}}{{\mathrm{Rrs}}_{m}}\right)/N$$

where Rrs_p_ refers to the water leaving reflectance calculated based on images, and Rrs_m_ refers to the camera band equivalent water leaving reflectance derived from the field spectrometer measurements. N refers to the number of sampling stations.

**Figure 6:**
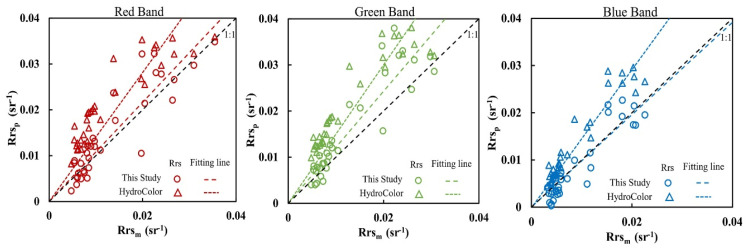
Scatterplots of water leaving reflectance derived from digital images at 31 sampling stations (using the method in this Study vs. the method in HydroColor) and measured by the spectrometer in RGB (red, green, blue) bands.

The digital images of water, sky, and multiple reflectance reference cards acquired at the 31 sampling stations were used to calculate Rrs by the method described in Section 3.3. The scatterplots of the image derived Rrs and spectrometer measured Rrs are shown in Figure 6; the validation parameters of RMSE, MRE, R^2^, and ratio are displayed in Table 2. Figure 6 and Table 2 show that the digital image-derived Rrs results had good accuracy, with an R^2^ of 0.94–0.95, MRE of 27.6%–31.8%, and a ratio of 0.98–1.21.

As a comparison, the same digital images of water, sky, and multiple reflectance reference cards acquired at the 31 sampling stations were also used to calculate Rrs by the HydroColor method described in Section 3.4. The Rrs were calculated using Equation (5) and compared with the Rrs measured by spectrometer, as shown in Figure 6. The validation parameters (RMSE, MRE, R^2^, and ratio) are displayed in Table 2.

Table 2 shows that the Rrs derived by the HydroColor approach has a high R^2^ (0.93–0.97) with spectrometer-measured Rrs; however, other accuracy parameters are not good (MRE of 58.7–86.1%, ratio of 1.41–1.50). Figure 6 shows that the Rrs derived by the HydroColor approach were almost all systematically overestimated, which explains the high R^2^ but low values of the other accuracy parameters. In comparison with the Rrs derived by the HydroColor approach, the Rrs derived by the approach in this study has higher accuracy.

Besides, fitting lines of the Rrs_p_ results by the methods both in this study and in HydroColor are plotted in each sub graph in Figure 6. It can be seen that the fitting lines of this study are all closer, 1:1 line, than those of HydroColor, which further shows that the Rrs derived by the approach in this study has higher accuracy.

Although the overall accuracy of the Rrs obtained by the camera measurements based on the method in this study is good, it can be seen from Figure 6 that the derived Rrs still scatter around the 1:1 line. The possible main reasons are: (1) the water body areas captured by the camera are not exactly the same as those captured by the field spectrometer; (2) four reference cards were used to fit the nonlinear response of the DN value with the reflectance, which still cannot completely avoid fitting error; (3) the digital image is digitalized in 8 bit and has only 256 gray levels. The reflectance of water is usually low, leading to low DN values in the image, which will be easily affected by the camera noise. These errors are difficult to be avoided completely. This shows that the calculation of water leaving radiation based on digital camera has limitations; although the overall accuracy is acceptable, it still cannot reach the accuracy of a field spectrometer.

## 4. Discussion

### 4.1. Comparison with the Theoretic Basis in HydroColor

**Figure 7:**
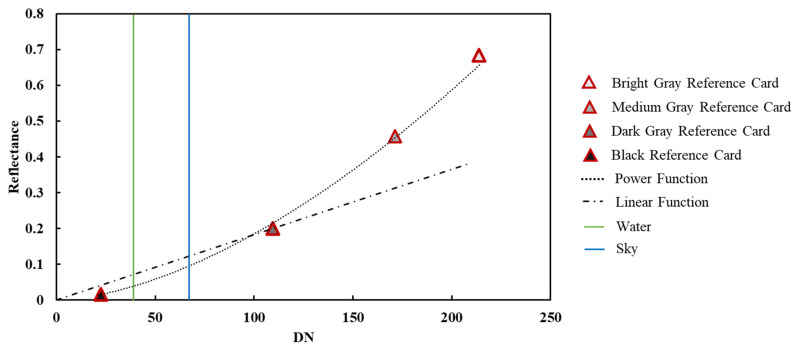
Comparison of the simulations of the relationship between digital pixel number (DN) and reflectance by HydroColor and this study. HydroColor utilizes one gray card to simulate a linear function between DN and reflectance; this study utilized four different reflectance reference cards to simulate a power function between DN and reflectance.

We further discuss the fundamental difference between the approaches of HydroColor and this study using the data of a sampling station in the Yuqiao Reservoir as an example. The red band DNs of the four reflectance reference cards, water, and sky are shown in Figure 7. The dot–dash curve is the power function simulated by the four reflectance reference cards, which is the basis of the approach in this study. The dot–dash line is the linear function simulated by the dark gray reference card alone, which is the basis of HydroColor approach.

The two vertical lines in Figure 7 represent the DN values of the water body and sky. Evidently, the water and sky reflectance calculated by the linear function are much higher than those calculated by the power function. The overestimation of water reflectance will lead to the overestimation of water leaving reflectance, whereas the overestimation of sky reflectance will lead to the underestimation of water leaving reflectance. However, the overestimation of sky reflectance can be counteracted by multiplying the low reflectance of skylight on the water surface. As a result, the overestimation of water and sky reflectance will lead to the overestimation of water leaving reflectance.

The reflectance of most water is lower than the reflectance of the 18% dark gray reference card and will be overestimated by the linear function simulation between the reflectance and DN. This can explain the systematic overestimation of Rrs derived by HydroColor approach (Figure 6).

### 4.2. Applicability of the Proposed Water Leaving Reflectance Derivation Method

With the advantages of low cost, real-time and rich information, citizen science data obtained by intelligent equipment are becoming an important source of water quality monitoring data. This represents a new direction in the field of water quality monitoring. In this study, we developed a low-cost and easy-to-use data method for quantitative inversion of water leaving reflectance from digital images. Compared with the simple linear fitting method adopted by HydroColor, multiple reflectance reference cards are used to correct the water leaving reflectance. Theoretically, our method is more in correspondence with the optical response of digital camera, and therefore more accurate water-leaving reflectance can be obtained. The high-precision water leaving reflectance data can be further used to retrieve some water quality parameters. With the water quality parameter information, a simple water quality evaluation of the water body can be made by using the citizen science data. More importantly, the water-leaving reflectance data and water quality parameter data obtained by this method can be better combined with the data obtained from satellite remote sensing, which can further support the joint application of the two data sources in water quality monitoring. Therefore, by filling the gap between citizen science data and other data sources, this quantitative method is essential for using and integrating citizen science data into water quality monitoring to improve data quality and expand monitoring networks.

The method developed in this study can be further adopted and programmed into smartphone Apps, after simple training for interested volunteers, it can help to take water surface photos of more places, to obtain more water quality parameters in a wider area, provide information for decision-making departments, and let citizens be environmentally conscious. However, it is notable that this method at this stage cannot be applied to fully automatic digital cameras and smartphones that cannot alter the exposure parameters manually. The uncertainties in digital images taken by automatic digital cameras and smartphones remain to be evaluated in the future, if this method is adapted into new automatic equipment. In addition, the method requires at least three reference cards with different reflectance, which is a little bit more costly than the one-card method, thereby possibly impacting the feasibility of large-scale promotion of this method. It is anticipated that the main users of this method will be researchers, environment protection workers, and volunteers.

## 5. Conclusions

In this study, we analyzed the optical characteristics of digital cameras to establish a water leaving reflectance measurement and correction method based on digital images. The method was applied to digital images captured with a Canon 50D digital camera from sampling stations in typical inland waters for calculating the water leaving reflectance. The results were validated using synchronously measured water leaving reflectance from a field spectrometer. The main findings of this study are as follows:

- When taking a photo of a particular object under the same illumination conditions, the change in the DN of digital images with increasing exposure time (fixed ISO and aperture) is nonlinear. Therefore, when the exposure time of the reference card, water, and sky images vary, they cannot be normalized to the same exposure time, and hence cannot be compared. Therefore, to calculate the remote sensing reflectance of water from the images, the same exposure parameters must be utilized to photograph the reference card, water body, and sky;
- The DNs of different reflectance objects in digital images are nonlinear with the reflectance. Therefore, it is not possible to use one reference card to calculate the DNs of a water body as its reflectance. At least three different reflectance reference cards are required to fit the DN and reflectance curve to accurately calculate the reflectance of various objects in an image;
- Based on the digital camera optical response analysis results, photography, and calculation methods suitable for water leaving reflectance were determined. First, the exposure parameters were fixed, and a variety of images of reflectance reference cards, water, and sky were taken. Then, by fitting the curve function relationship of the reflectance using multiple reflectance reference cards and DN, the water and sky reflectance and, subsequently, the water leaving reflectance were calculated.
- The proposed method was applied to photograph water, sky, and reflectance reference cards at 31 sampling stations of Taihu Lake and the Yuqiao Reservoir to calculate the water leaving reflectance. Compared with Rrs measured synchronously with a spectrometer, the R^2^ values of the red, green, and blue bands were 0.94, 0.95, 0.94, the mean ratios were 1.08, 1.21, and 0.98, and the average relative errors were 27.6%, 29.8%, 31.8%, respectively. In comparison, the remote sensing reflectance calculated by the HydroColor approach (i.e., using one reference card) was systematically overestimated;
- The main advantage of this proposed approach is that it is based on the optical response of a digital camera and has higher accuracy in deriving water leaving reflectance from digital images. The main disadvantages are that it cannot be applied to fully automatic cameras, which cannot control exposure parameters, and the cost of using at least three different reflectance reference cards; these issues may impact on the large-scale promotion of the method. However, the method presented here provides an improved theoretical foundation for quantitative water quality monitoring using digital and smartphone cameras and further provides an easy and useful tool for researchers, environment protection workers, and volunteers.

## Figures and Tables

**Table 1:** Water surface experiment metadata.

Water Body Name	Center Longitude	Center Latitude	Experiment Date	Time Range (Local Time)	Sampling Number
Taihu Lake	120.02° E	31.17° N	2019-05-01	9:30–15:45	9
Yuqiao Reservoir	117.53° E	40.03° N	2018-11-01	9:55–13:50	13
Yuqiao Reservoir	117.53° E	40.03° N	2018-11-22	10:15–14:15	9

**Table 2:** Accuracy evaluation of water leaving reflectance derived from digital images acquired at 31 sampling stations using the methods proposed in this study and the HydroColor smartphone application.

Band	RMSE (sr^−1^)		MRE (%)		R^2^		Ratio	
	This Study	Hydro-Color	This Study	Hydro-Color	This Study	Hydro-Color	This Study	Hydro-Color
R	0.0044	0.0087	27.6	81.9	0.94	0.93	1.08	1.41
G	0.0053	0.0092	29.8	86.1	0.95	0.95	1.21	1.50
B	0.0028	0.0056	31.8	58.7	0.94	0.97	0.98	1.46
